# Identifying inaccuracies in gene expression estimates from unstranded RNA-seq data

**DOI:** 10.1038/s41598-019-52584-w

**Published:** 2019-11-08

**Authors:** Mikhail Pomaznoy, Ashu Sethi, Jason Greenbaum, Bjoern Peters

**Affiliations:** 1 Division of Vaccine Discovery, La Jolla Institute for Immunology, La Jolla, CA United States; 2 Department of Medicine, University of California San Diego, La Jolla, CA United States

**Keywords:** Software, Data processing

## Abstract

RNA-seq methods are widely utilized for transcriptomic profiling of biological samples. However, there are known caveats of this technology which can skew the gene expression estimates. Specifically, if the library preparation protocol does not retain RNA strand information then some genes can be erroneously quantitated. Although strand-specific protocols have been established, a significant portion of RNA-seq data is generated in non-strand-specific manner. We used a comprehensive stranded RNA-seq dataset of 15 blood cell types to identify genes for which expression would be erroneously estimated if strand information was not available. We found that about 10% of all genes and 2.5% of protein coding genes have a two-fold or higher difference in estimated expression when strand information of the reads was ignored. We used parameters of read alignments of these genes to construct a machine learning model that can identify which genes in an unstranded dataset might have incorrect expression estimates and which ones do not. We also show that differential expression analysis of genes with biased expression estimates in unstranded read data can be recovered by limiting the reads considered to those which span exonic boundaries. The resulting approach is implemented as a package available at https://github.com/mikpom/uslcount.

## Introduction

**Figure 1:**
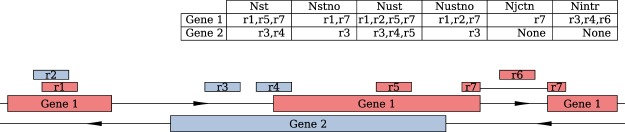
Illustration of count types described in the main text. Color of boxes reflects the strand. Table on top explicitly enumerates which reads are counted for every count type.

RNA sequencing assays have become the default choice for transcriptomic studies due to their high sensitivity, dynamic range, and no need of prior knowledge of a sample’s nucleotide content. Due to the common procedure of RNA-seq library preparation by ligating adapters to double stranded DNA, the information about the RNA’s strand of origin in the sample may be lost. In this case it becomes hard to unambiguously assign a read to a particular gene if the gene has an opposite strand counterpart overlapping its exons (e.g. see read 5 in Fig. 1). Moreover, even in the lack of exonic overlap, if two genes are in close proximity, “carry-over” expression may occur if one of the genes is highly expressed^1^. Problems arising from unstranded assays have been highlighted in several previous studies (e.g.^1–3^), and strand-specific protocols have been developed ^4,5^ that avoid this problem altogether. However, despite known advantages of stranded data, many currently generated RNA-seq datasets are prepared in a non-strand-specific fashion (typically because this enables working with smaller sample volumes), and no bioinformatic approaches have been developed to specifically identify where unstranded data could be problematic.

In this work we utilized a stranded RNA-seq dataset generated from sorted cell types found in peripheral blood that was recently published in the DICE database^6^. We investigated how strand-aware analysis vs. strand-unaware analysis (ignoring present strand information) impacted the expression estimates for individual genes, and how reproducible those findings were across cell types. Based on these analyses, we found a set of parameters that could identify genes that are likely erroneously quantitated in unstranded data. We integrated these parameters into a novel prediction algorithm that provides an estimate of how likely a gene’s count is compromised by transcription on the opposite strand in unstranded data. The algorithm is available as a Python package which assigns sequencing reads to features and can be incorporated for high-throughput sequencing analysis of strand-specific and non-strand-specific RNA-seq libraries.

## Results

### A substantial fraction of genes has mis-estimated expression levels in unstranded data

We used stranded RNA-seq data of 15 sorted cell types from the DICE database for a systematic comparison of stranded vs. unstranded expression estimates. Raw reads of 10 samples of each cell type were aligned to the genome using STAR aligner ^7^. To investigate the impact of strand information on gene expression estimates, we assigned the aligned reads to gene features with- and without that information. We followed the commonly used “exon union”-based approach as implemented in HTSeq package framework^8^, and implemented it into an in-house software package (uslcount). As shown in Supplementary Fig. 1, our implementation produces essentially identical results to those obtained with HTSeq. Our developed framework generates strand-aware, strand-unaware gene counts as well as other metrics required for downstream analysis (see Fig. 1 for illustrative explanation):

1. N_st_ – stranded counts; it is regularly used for the analysis of stranded datasets.
2. N_stno_ – stranded counts in non-overlapping regions (excluding overlaps with other genes).
3. N_ust_ – unstranded counts (in full gene span, including overlaps with other genes).
4. N_ustno_ – unstranded counts in non-overlapping regions; it is regularly used for the analysis of unstranded datasets.
5. N_jctn_ – counts of reads spanning exon-exon boundaries (aligned with splice junctions).
6. N_intr_ – counts of reads overlapping intronic regions.

To identify genes that could be erroneously quantitated with an unstranded assay, we used a log_2_(N_ustno_/N_stno_) which is the log-ratio of unstranded vs. stranded counts of reads mapped within the genes’ unique regions (not overlapping with other genes). N_stno_ is used but not N_st_ because the former accounts for reads in the same region as N_ustno_ but the latter accounts for reads in a larger span if gene overlaps with the other gene. This can falsely reduce the value of log_2_(N_ustno_/N_stno_). Also important to note that by definition N_stno_ ≤ N_ustno_ so log_2_(N_ustno_/N_stno_) is non-negative. We considered an unstranded gene count as biased if this log_2_-ratio is greater than 1 (more than two-fold overestimation) and refer to these genes as strandedness-affected.

**Figure 2:**
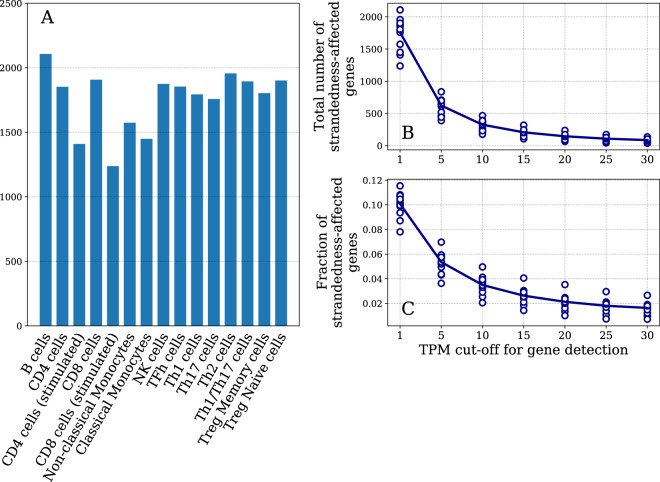
There is a significant number of genes erroneously quantitated in unstranded datasets. (**A**) Number of strandedness-affected genes is various cell types. (**B,C**) absolute count and fraction of strandedness-affected genes respectively. X-axis represents TPM cut-off threshold used to identify detectable genes. Every circle corresponds to a cell type for a particular cut-off.

Overall numbers of genes with biased gene expression (strandedness-affected) for each of 15 investigated blood cell types are shown in Fig. 2. On average 1,758 detectable genes (mean of all cell types; including non-protein-coding species) had a value of log_2_(N_ustno_/N_stno_) > 1 (Fig. 2A). This constitutes 10.1% of genes (mean of all cell types) with detectable expression (TPM_unstranded_ > 1). 3,801 detectable genes were strandedness-affected in at least one cell type of which 687 are protein-coding (see Supplementary Table 1 for the full list of genes).

We further looked into genes for which log_2_(N_ustno_/N_stno_) > 1 in a majority of investigated cell types and focused on those with the highest observed expression. These genes would have seemingly high expression in unstranded datasets, but that would be due to transcription on the opposite strand. Among these genes we found several important genes related to immune response (the focus of the DICE database that we were using), such as CXCR6, CD244, IL3RA, IL17RB; signaling related genes e.g. RHOQ, MAP3K13; cytochromes CYP2D6, CYP3A5 as well as other genes of high interest for transcriptomic analysis (see Supplementary Table 1). We were also surprised to see the mitochondrion encoded gene MT-ND6 on top of genes with the highest distorted expression estimates. It turned out that it is the only protein-coding gene located on the L-strand of the mitochondrial DNA. H-strand of mitochondrion is actively transcribed leading to overestimated expression of MT-ND6.

Next, we investigated the overlap of strandedness-affected genes identified in different cell types. We found that these genes substantially overlap. Specifically, Jaccard similarity coefficient (ratio of intersection vs. union of strandedness-affected genes in two cell types) is equal to 0.48 when comparing B cells and naive CD4 cells. When comparing more similar cell types like Th1 and Th17 Jaccard similarity is even higher and equals to 0.78. However, we were able to find genes which expression is compromised in one cell type and not the other 14 cell types. For example, we were able to find 242 genes prone to opposite strand bias exclusively in B cells (the most “distant” from other cell types in our dataset; see Supplementary Fig. 2). For other cell types number of unique strandedness-affected genes was smaller.

Given that “leaky” expression of a gene beyond its boundaries that is incorrectly mapped to a gene on the opposite strand is generally low, we hypothesized that genes with low intrinsic expression level were more likely to be impacted by it. Indeed, the number of strandedness-affected genes was reduced for all cell types when the expression detection cut-off was increased (Fig. 2B). Moreover, not only the absolute numbers, but number of strandedness-affected genes as a fraction of all observed genes decreased substantially (Fig. 2C). This happens because reads originating from the opposite strand are usually in lower quantities than “true” reads belonging to moderately or highly expressed genes. Hence, these “wrong” reads are less likely to affect genes with high expression but are enough to result in substantial overestimation of genes with low expression.

### Lack of strand information affects differential expression (DE) analysis

A common question addressed by transcriptomic experiments is the identification of genes differentially expressed between two conditions. We tried to identify how strand information affects DE analysis with DESeq2 and used stranded-aware and strand-unaware counts to find differences between relatively distant cell types (T_H_1 CD4 cells vs B cells), moderately distant cell types (T_H_1 CD4 cells vs NK cells) and similar cell types (T_H_1 CD4 cells vs T_H_17 CD4 cells). We considered DEGs identified using stranded counts as “true”. Relative to that DEGs obtained using unstranded counts were considered as false positives if they were not differentially expressed in stranded analysis and as true positives if they are differentially expressed in stranded analysis. Genes differentially expressed in stranded analysis and not differentially expressed in unstranded analysis were considered as false negatives.

**Figure 3:**
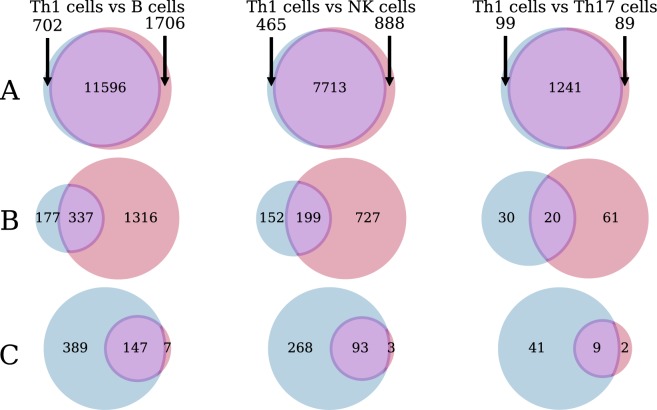
Comparison of DEGs called on strand-aware and strand-unaware counts. Three columns of Venn diagrams correspond to three comparisons of cell types indicated on top. Left circle (blue) always corresponds to “true” DEGs identified with strand-aware counts. Top row (**A**) shows overlap of DEGs obtained with strand-aware vs. strand-unaware counts. Middle row (**B**) is the same but only for strandedness-affected genes (those where log_2_(N_ustno_/N_stno_) > 1). Bottom row (**C**) covers the same genes that are prone to expression overestimation used in B, but uses junction counts for differential expression analysis.

Overall number of DEGs in the three comparisons is concordant with expected inter-cell type differences with the largest number of DEGs observed in T_H_1 vs B cells comparison and the smallest in T_H_1 vs. T_H_17 cells comparison. For every comparison we observed false positive DEGs: approximately 10% of all DEGs called in strand-unaware analysis were false positives (see Venn diagrams in Fig. 3A). Similarly, we observed a fraction of false negative DEGs in strand-unaware analysis, specifically 6.56% of “true” DEGs are false negatives in analysis with strand-unaware counts (average of three comparisons in Fig. 3A).

We next tried to identify how this erroneous differential expression calls are related to opposite strand transcription bias. We quantitated number of false positives and false negatives within genes with log_2_(N_ustno_/N_stno_) > 1, i.e. those affected by transcription from the opposite strand. Relative numbers of false positives and false negatives is substantially higher within strandedness-affected genes. Specifically, 77% of all DEGs called using strand-unaware counts were false positives (0.77 false positive rate, average of 3 comparisons) and 46% of all “true” stranded DEGs were false negatives (0.46 false negative rate, average of 3 comparisons, see Fig. 3, row B) within the strandedness-affected genes. Also notably the majority of false positives in Fig. 3A can be explained by this bias: 75.8% of false positives belong to strandedness-affected genes (average of three comparisons).

We were also interested how bioinformatics pipeline may affect these observations. To do so we performed the same analysis using a different, transcript-based approach. W quantitated genes using RSEM (with bowtie aligner) and performed differential expression analysis using edgeR. In this case we observed slightly higher rates of false positives (15.9% on average of three cell type comparisons compared to 10% for STAR + DESeq2 analysis) and false negatives (13.2% compared to 6.56% for STAR + DESeq2 analysis, Supplementary Fig. 3). However less of these miscalls can be explained by our definition of strandedness-affected genes. 25.7% of false positives belong to strandedness-affected (those for which log_2_(N_ustno_/N_stno_) > 1) genes (vs. 75.8% for STAR + DESeq2 analysis).

### Read alignments of genes with biased unstranded counts are different from other genes

We investigated if it is possible to identify genes that had incorrect expression estimates in unstranded data by examining the read alignments for genes with very high values of log_2_(N_ustno_/N_stno_), i.e. those where the majority of reads attributed are in fact due to the opposite strand expression. Manual inspection of these alignments revealed several characteristics which stood out from other genes, and made intuitive sense: alignments with reads originating from the opposite strand will ignore the gene’s exon-intronic structure. Because of that, the number of observed reads spanning splice junctions is lower than expected for a regular gene while the number of reads in intronic regions is higher than expected. In addition, we frequently found a highly expressed gene on the opposing strand, whose transcription carried-over and is presumably the source of reads detected. Based on each of these observations, we proceeded to identify metrics that would allow us to identify genes that might be affected by expression on the opposing strand based on examining the alignments alone if only unstranded information is available.

#### Number of detected splice junctions

**Figure 4:**
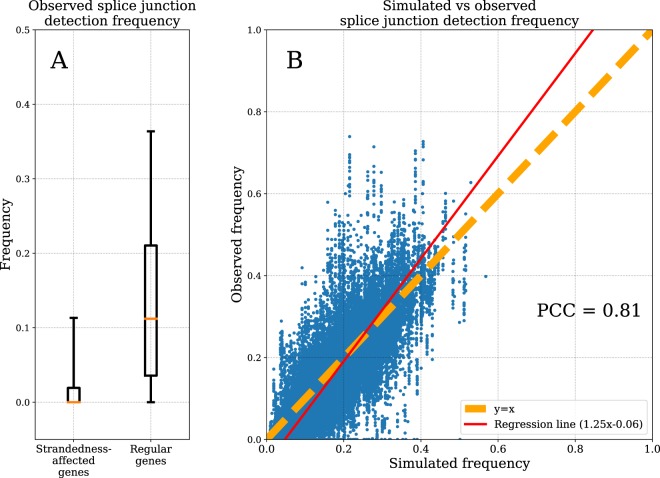
Reads spanning splice junctions as a predictor of expression estimation bias. (**A)** Box-plot for observed probability of splice junctions in regular and strandedness-affected genes. Box denotes inter-quartile range, orange line is the median; bottom and top caps denote 5^th^ and 95^th^ percentiles correspondingly. (**B)** Comparison of *in* *silico* simulated splice junction detection probabilities (X axis) and observed frequency of splice junction detection (Y axis). Points for random sample of 3,000 genes and all 15 cell types are shown.

If a gene’s locus is substantially affected by expression from the opposite strand, then the number of detected splice junctions in the read alignment is expected to be lower than that expected for other genes with the same expression level (see Supplementary Fig. 4 for an extreme example). As a metric to account for that, we compared the number of junction reads as a fraction of the unstranded count N_jctn_/N_ust_ (see Fig. 1). Indeed, the median of this ratio is 0.11 for genes not compromised by opposite strand expression while it equals 0.0 (mean 0.02) for strandedness-affected genes (Fig. 4A).

This striking difference in observed frequency of spliced reads suggests to use it as a predictor of strandedness-affected genes. However, for some genes number of detected reads spanning exon boundaries can be intrinsically low due to properties of exon-intronic structure. This frequency also depends on data generation characteristics, more specifically, read length, read configuration (single- or paired-end) and insert size (for paired-end reads^9^). To calculate the number of reads spanning exon-exon junctions expected for a particular gene in a particular read configuration we used *in silico* simulation using the following assumptions:

1. Transcript isoforms are equally expressed, i.e. number of detectable RNA molecules is the same for every gene’s transcript isoform.
2. Transcript coverage is even along its length. Computationally it means that probability of detecting a read covering certain portion of a transcript is the same regardless of the read start position within the transcript.

The first assumption might be too strong if genomic annotation used for simulation contains rare transcripts (e.g. uncommon transcripts with retained introns). To minimize effect of this bias annotations should not contain very rare transcript models. The second assumption might be too strong for genes where coverage is skewed in some portions of gene’s exonic span, for example due to intrinsic bias in sequencing technology.

To see how prediction based on above-mentioned assumptions recapitulates real data we simulated expected splice junction probabilities for all the genes present in Gencode V28 annotations (basic transcript set). Using available stranded dataset we calculated “real” or observed frequency of reads spanning known exon-exon junctions. Then we compared these observed frequencies with *in silico* simulated frequencies for each gene in all investigated cell types. Scatter plot for observed vs. predicted splice junction probabilities is shown in Fig. 4B. Some of the genes significantly deviated from equality of predicted and observed frequencies. These might be those for which above-mentioned assumptions do not hold. Nevertheless, we observed very high overall concordance (Pearson correlation 0.81) between predicted and simulated frequencies of junction reads.

Since it is possible to estimate how many splice junctions are expected for a particular gene model, it is possible to construct metric for distinction of strandedness-affected genes. Specifically a log-ratio of the number of observed vs. the number of expected (*in silico* simulated) splice junctions can be used as a predictive metric. Genes with low value of this ratio are more likely to be affected by opposite strand bias.

#### Intronic reads

**Figure 5:**
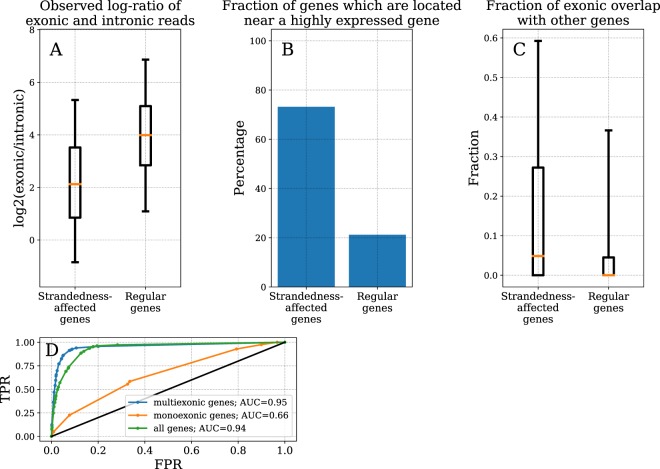
Read alignment characteristics and prediction performance. (**A**–**C**) Alignment characteristics in strandedness-affected genes, i.e. those for which log_2_(N_ustno_/N_stno_) > 1 and genes with unbiased expression. (**A)** Box-plot for log-ratio of normalized exonic vs. normalized intronic reads. (**B**) Bar plot for fraction of genes located proximally to a highly expressed gene (TPM > 40). (**C**) Box-plot of fraction of exonic span overlapping with other genes’ exons. A and C: Box denotes inter-quartile range, orange line is the median; bottom and top caps denote 5^th^ and 95^th^ percentiles correspondingly. (**D**) AUC analysis for prediction of strandedness-affected genes in B cells using prediction models trained on other cell types.

The typical pattern of RNA-seq read alignment coverage is characterized by spikes within exonic regions of a gene and drops of coverage within intronic regions. If many reads from the opposite strand are aligned to a gene’s locus, this structure is distorted and coverage of intronic regions can become comparable to that of exonic regions. To quantify that, we calculated the number of intronic (N_intr_) and exonic reads (N_ustno_) for a given gene and normalized them to the lengths of intronic and exonic regions of the gene correspondingly. As a metric, we examined the log-ratio of normalized exonic vs. normalized intronic reads in regular genes and strandedness-affected genes. We found that for regular genes this log-ratio has a median of 3.98 while for strandedness-affected genes it is substantially lower with a median of 2.12 (Fig. 5A).

#### Presence of highly expressed genes on opposing strand

To determine if presence of a highly expressed gene on the opposite strand was predictive of erroneous quantification of gene’s expression, we quantified genes which had a gene on the opposite strand within 2,000 bp that is highly expressed. We found that 73.1% of the strandedness-affected genes were located near a gene with TPM > 40 and only 21.3% of regular genes were located near a gene with expression level higher than 40 TPM (Fig. 5B).

For prediction purposes we scan every gene for its neighboring genes (not further than 2,000 bp from the gene inspected) and compute N_ust_ count normalized to gene length. Then maximum of normalized counts of the neighboring genes is used as a predictive metric.

#### Overlap with other genes

Finally, we were interested if the fraction of gene’s exonic span overlapping with some other gene also contributes to likelihood of expression bias. We found that the majority of regular as well as strandedness-affected genes do not have exonic overlap with other genes. Nevertheless, exonic overlap is more likely for strandedness-affected genes. More specifically, 75th percentile of exonic overlap equals to 0.27 for strandedness-affected genes, i.e. for 25% of these genes at least 27% of their exonic span overlaps with exons of other gene. For regular genes 75^th^ percentile of exonic overlap fraction is much smaller averaging 0.045 (see Fig. 5C).

### Using machine learning to predict strandedness-affected genes

Next we used the defined metrics to predict genes affected by expression on the opposite strand using machine learning. We used decision trees for constructing prediction models. We also tried neural network approach but with no increase in performance, possibly because available number of features is too low for this technique. As 4.9% of all protein-coding genes (38.9% of all genes) are monoexonic, usage of reads spanning exon boundaries and intronic reads is not possible so number of available metrics is lower (only two) for monoexonic genes.

We divided 150 samples of our dataset (15 cell types x 10 donors) into 140 samples of a training set (excluding B cell samples) and 10 samples of a test set (10 B cells samples). B cells were picked for testing because of their relatively high distance from other 14 cell types. Two separate decision trees were trained for multiexonic and monoexonic genes. Next we used these trained decision trees to predict strandedness-affected genes in B cells. The quality of this prediction was estimated with AUC analysis. Figure 5D shows resulting ROC curves for multiexonic and monoexonic genes separately, as well as summary curve for both classes of genes combined. For multiexonic genes we managed to reach AUC value of 0.96. For monoexonic genes two powerful prediction metrics are not applicable, nevertheless prediction based on other two metrics is still substantially better than random guess (0.63) though much smaller than for multiexonic genes. Combined ROC curve for all genes reached AUC value of 0.94.

### Detected splice junctions as a measure of gene expression

As already discussed above, reads spanning known exon boundaries are very unlikely to originate from the opposite strand. For example, read 7 in Fig. 1 almost certainly originates from gene 1’s strand and not the opposite strand. This happens because RNA splicing machinery targets a certain RNA strand but not its reverse complement. Indeed introns contain conserved 5’GT-(N)_n_-AG-3′ motif ^10^ which does not give functional splicing site when reverse complemented. Additionally, other RNA motifs are also involved in spliceosome recognition, further ensuring strand specificity of a splicing event. Because of that we reasoned that the number of junction reads can be used as an expression proxy in cases where expression is compromised by the opposite strand bias.

To validate this approach we performed a differential expression analysis for three comparisons discussed above (T_H_1 CD4 cells vs B cells, T_H_1 CD4 cells vs NK cells, T_H_1 CD4 cells vs T_H_17 CD4 cells) using junction counts instead of strand-unaware counts. DEGs identified in this way were compared to DEGs resulting from strand-aware counts (“true” DEGs). As was shown in Fig. 3B using strand-unaware counts results in significant numbers of false positives and false negatives among the strandedness-affected genes. When we performed differential expression analysis on the same set of strandedness-affected genes but using junction counts, performance of the analysis improved significantly (Fig. 3C). In this case we still observed substantial number of false negatives but the number of false positives (false positive rate) was reduced to 7% (average of three comparisons). Observed false negatives can be explained by fewer absolute numbers of junction counts compared to regular unstranded counts resulting in lower statistical power.

### Identification of strand bias in public datasets

We were interested if strand-specificity related errors might have an impact on some of the publicly available datasets. As an example, we downloaded raw reads for a subset of Human Protein Atlas^11^ (HPA) data and applied our prediction model to find potential errors of quantification related to expression from the opposite strand. We analyzed alignments of 32 human tissues available in HPA. We then investigated the genes which are predicted to be strandedness-affected with probability greater than 0.5. We observed from 0.45% to 3.72% of genes to be potentially biased in 32 profiled tissue samples. Manual inspection of this cases reveled some false positives as well as several examples of potentially erroneous gene quantitation which can be explained by transcription from the opposite DNA strand.

For example, opsin 1 gene (OPN1SW) encodes a photoreceptor pigment protein responsible for recognition of short wave-length part of visible spectrum. According to HPA its transcription is detectable in various human tissues including gallbladder and thyroid gland^12^. Expression of OPN1SW in those compartments seemed biologically unlikely. Indeed, antibody staining of gallbladder tissues (available in HPA) did not detect opsin 1 expression in gallbladder. Manual inspection of read alignments in OPN1SW gene locus clearly demonstrates that RNA-seq detected expression can be explained by the reads arising from the opposite strand. Those reads can be attributed to proximally located and highly expressed calumenin (CALU) gene (see Supplementary Fig. 4).

As another example, we found discrepancies in expression of GPR17 gene. This gene is located in the intronic region of LIMS2 gene encoded in genomic strand opposite to GPR17′s strand. According to HPA, RNA of this gene is detected in many tissues including rather significant expression in the lung (TPM 7.3)^13^. On the other hand, antibody stainings available in HPA did not indicate GPR17 expression in the lung. Manual inspection of the read alignments (see Supplementary Fig. 5) hints that reads contributing for observed expression of GPR17 are likely to originate from the nascent RNA of LIMS2 gene.

### Implementation

Developed predictions model is implemented in the Python package named “uslcount” (**U**n**S**tranded **L**ibrary **Count**). The package has three entry point tasks used in the pipeline:

1. **Build**. This step creates a genomic database which can be used for other steps. Pre-formatting of the genomic files saves time on next read counting invocations. At this step genomic data from GTF file is used to construct gene models stored in plain text data structures.
2. **Count**. This task assigns reads of a supplied BAM file to the gene features stored in the genomic database created at the previous step.
3. **Analyze**. This task should be applied to unstranded libraries only. It does read counting task performed by **count** and additionally it outputs confidence score ranging from 0 to 1 estimating how likely gene’s expression is overestimated due to transcription from the opposite strand. 1 means least likely (high confidence in expression), 0 means very likely (low confidence in expression). Also junction counts for each gene are provided in the output.

Count task is applicable to strand-specific as well as non-strand-specific RNA-seq data and can be used in a high-throughput sequencing analysis pipeline to obtain raw gene counts. We compared the performance of uslcount with other existing packages for counting reads at gene level such as htseq-count 0.11.2 (from HTSeq library ^8^), featureCounts 1.6.3, summarizeOverlaps (from GenomicAlignments 1.18.0 R package ^14^) and the STAR aligner’s read counting functionality. These packages employ similar approach of assigning reads to gene features and hence the results obtained are very similar except for summarizeOverlaps (Supplementary Fig. 8). Uslcount package demonstrates very similar results to htseq-count (Supplementay Fig. 1), featureCounts (Supplementary Fig. 6) and STAR (Supplementary Fig. 7). Uslcount is faster than htseq-count but took slightly longer than featureCounts and summarizeOverlaps in the benchmarks (Supplementary Fig. 9).

## Discussion

It is well known that strand-specific RNA-seq library preparation protocols are more accurate for gene expression quantification than non-strand-specific. Uncertainties in read strand assignment can substantially skew expression estimate for some genes, and is a problem for some genes such as OPN1SW and GPR17 as shown above. While this issue has been widely acknowledged, no algorithmic approach has been developed so far to identify which specific genes are at risk of being mis-interpreted.

We here utilized stranded data of 15 blood cell types available in DICE-DB to further investigate errors dependent on strandedness of RNA-seq data. We found that a significant fraction of all genes are prone to substantial (over two-fold) overestimation of expression due to transcription from the opposite strand. Specifically, strand-unaware counts are unreliable for 10.1% of all genes (Figs. 2A) and 2.5% of protein-coding genes for a given blood cell type. Such strandedness-affected genes from one cell type majorly overlap with those in the other cell type. However, every cell type has a set of its specific strandedness-affected genes. Furthermore, expression overestimation is dependent on sequencing read length and configuration. This pointed us to search for a method to detect read counting bias based on sequencing read alignments.

It was previously shown that lack of strandedness information may affect downstream analysis^3^. In particular differential expression analysis is significantly affected generating false positives and false negatives compared to “true” analysis derived from stranded data. We observed similar results in DICE-DB data using our STAR + “htseq-count” + DESeq2 pipeline (actually our implementation of the htseq-count counting strategy was used). Importantly we have shown that the majority of false positives in such analysis are actually strandedness-affected genes (more than 75%). Slightly higher rates of false positives and false negatives were observed when we switched to another pipeline utilizing transcript-based counting (RSEM + edgeR).

We found that alignments of strandedness-affected genes have characteristics different compared to regular genes. Specifically, reads originating from the strand opposite to a gene’s strand ignore the gene’s exon-intronic structure. Additionally, it is more likely to find strandedness-affected genes proximally to or even overlapping with another gene. Based on this differences we developed a machine learning model to predict if the gene’s expression is overestimated due to transcription from the opposite strand. Validation of our model on B cell samples demonstrated AUC value of 0.94 in ROC-based performance analysis. Despite this rather high AUC value, we have to state that current FPR levels can still lead to substantial number of false positives because strandedness-affected genes are a minority of all genes. The fact that they constitute 2.5% of all protein-coding genes makes overall number of false positives and true positives comparable. Nevertheless, this model can be used to get an initial estimate of how likely gene is erroneously quantified while manual inspection of the alignment can be essential for confirmation at the gene level.

It is also important to note that here we analyzed single-end 50 bp read data while longer and paired-end reads are often available. We were interested to rule out if our model is over-fitted to read configuration of the training dataset and might under-perform on other datasets. On the contrary, we observed substantial improvement of prediction accuracy when applying the algorithm to paired-end 75 bp data (Supplementary Fig. 10). This can be explained by the fact that using longer reads significantly increases the probability of splice junction detection^9^. This can make overall prediction of strandedness-affected genes more robust as number of reads aligned with junctions is the most informative metric for our prediction model. It also highlights that for more robust quantitative transcriptomic profiling longer paired-end reads are beneficial, especially when unstranded data is generated. Reads aligned with junctions can be almost certainly assigned to particular strand and hence can give a more stable estimate of gene expression. As number of such reads increases for longer reads so decreases the ambiguity in read feature assignment for overlapping genes.

Finally, we tried to find a possibility to overcome uncertainties in differential expression analysis by constraining to only reads aligned with splice junctions. Indeed, we were able to demonstrate that using junction reads results in significant reduction of false positives compared to unstranded data. Number of such junction reads usually constitute a minority of all reads aligned to a gene’s locus. It makes differential expression analysis based on junction reads less powerful and hence leads to certain fraction of false negative results. Nevertheless, for longer sequencing reads their fraction increases substantially. It makes junction reads a more reliable alternative for a gene of interest if expression bias is confirmed for the gene.

Approach described in this work is implement in Python package “uslcount”. The package is capable of counting aligned reads obtained from unstranded or stranded data. It is applicable in a routine RNA-seq processing pipeline. Importantly, for unstranded data it can give additional information about strand biases which can prevent occasional misinterpretations in unstranded RNA-seq data analysis.

## Materials and Methods

### Read data used

Main dataset used for training and testing consists of FASTQ files of 15 cell types of 10 donors available from DICE-DB^6^. For investigating HPA public dataset we downloaded 32 FASTQ samples (1 sample per tissue) from ENA archive, study PRJEB4337. Additionally, for testing package performance on stranded paired-end data we used SRA archive SRR5424812.

### Read alignment and annotations

Raw FASTQ reads were aligned to genome using STAR aligner^7^ against GRCh38 genome with parameters STAR –runThreadN 2 –outFilterType BySJout –alignSJoverhangMin 8 –alignSJDBoverhangMin 1 –alignIntronMin 20 –alignIntronMax 1000000 –alignMatesGapMax 1000000. Gencode V28 (basic set) genomic annotation was used at alignment stage and downstream feature count assignments. STAR-derived alignments are used throughout the manuscript. Additionally, to compare with transcript-based counting approaches reads were quantitated against the same genome build and annotation using RSEM^15^ with parameter strandedness equal to “reverse” or “none” to get strand-aware and strand-unaware counts correspondingly.

Differential expression analysis between different the cell types was implemented using DESeq2^16^. Additionally we used edgeR^17^ in “classic” (no GLM functionality) mode to obtain differentially expressed genes.

### Plotting and visualization

Plots were constructed using Python Matplotlib package. Visualization of alignments was done using IGV genome browser^18^.

### Machine learning

For constructing decision tree we used DecisionTreeClassifier class from scikit-learn^19^ with max_depth parameter of 5 for multiexonic and 3 for monoexonic genes, i.e. 1 more than number parameters, was used for training.

### Comparison with other read counting software

For comparisons of counts and computational time benchmarking we used 10 samples of Treg cells from DICE-DB. Following parameters were used for read counting software packages: *featureCounts* -T 1 -a gtf_file -t exon -g gene_id -s 2 -Q 10; *htseq-count* -m union -r pos -t exon -i gene_id -s reverse -f bam; *summarizeOverlap*(mode = “Union”, singleEnd = T, ignore.strand = F, preprocess.reads = invertStrand); uslcount was invoked with parameters python3 -m uslcount count -strand R. These packages were tested on a CentOS 7 box with 48 Intel Xeon 2.20 GHz CPUs and 314 GB of memory. All programs were run using a single CPU core.

## Supplementary information

Supplementary Figures

Supplementary Dataset 1

## Data Availability

Repository with the source code of the developed package is hosted on GitHub and can be found here: https://github.com/mikpom/uslcount.
