# Supplementary material for: Identifying inaccuracies in gene expression estimates from unstranded RNA-seq data
Source: Sci Rep. 2019 Nov 8;9:16342. doi: 10.1038/s41598-019-52584-w (PMC6841694; doi:10.1038/s41598-019-52584-w)
Supplement: Supplementary file 1 — Supplementary Figures [file 41598_2019_52584_MOESM1_ESM.pdf]

Supplementary information

Identifying inaccuracies in gene expression estimates from unstranded RNA-seq data

Mikhail Pomaznoy[1], Ashu Sethi[1], Jason Greenbaum[1], Bjoern Peters[1,2]

[1]Division of Vaccine Discovery, La Jolla Institute for Immunology, La Jolla, CA.

[2]Department of Medicine, University of California San Diego, La Jolla, CA, United States.

Supplementary Figures 1-10.

Supplementary Table 1 is provided as a separate .XLSX file.

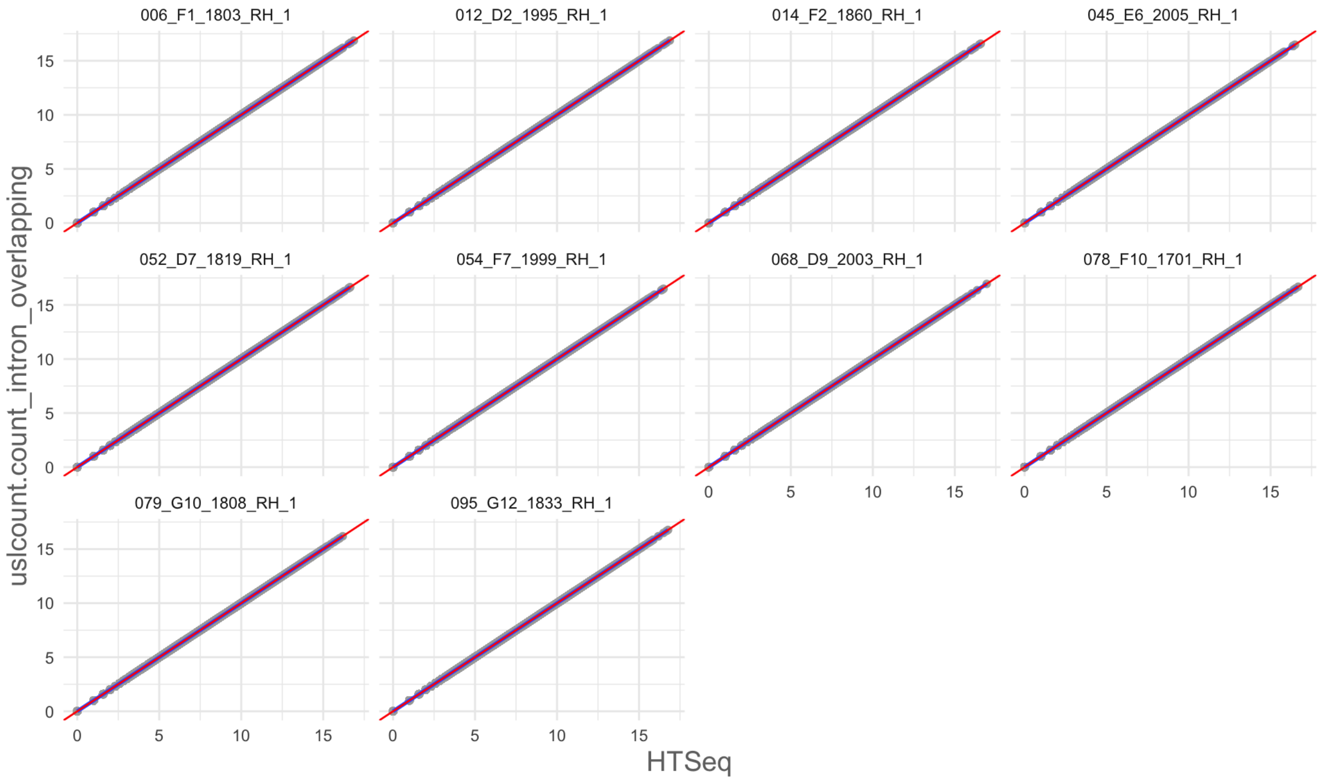

SFigure 1: Counts obtained with uslcount vs. HTSeq. *Scatter plot of counts obtained with uslcount package and htseq-count command from HTSeq package are shown. Very similar counts were observed for all 10 samples investigated.*

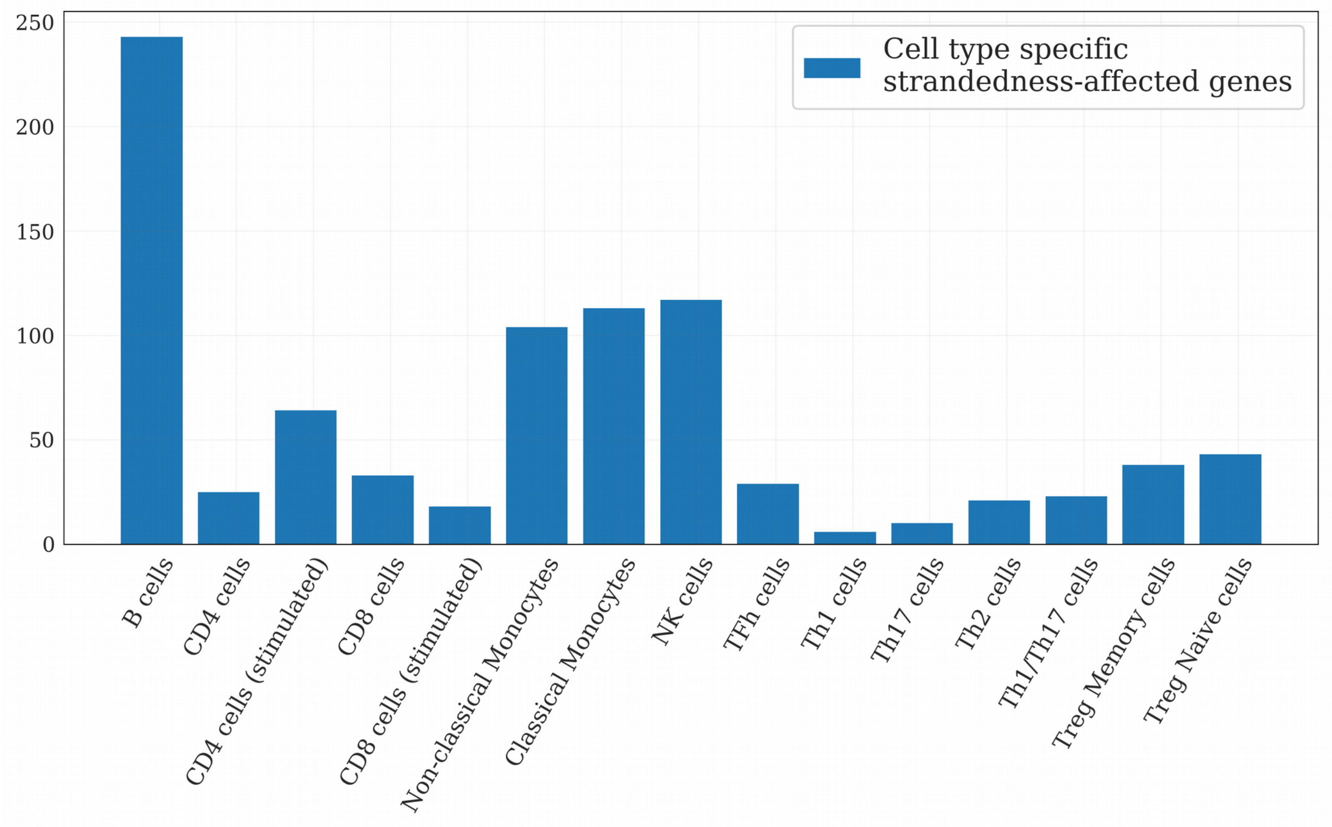

SFigure 2: Cell type-specific strandedness-affected genes. *Bar plot showing number of erroneously quantitated genes (for which log2(Nust/Nstno) > 1) uniquely in one cell type but not the others.*

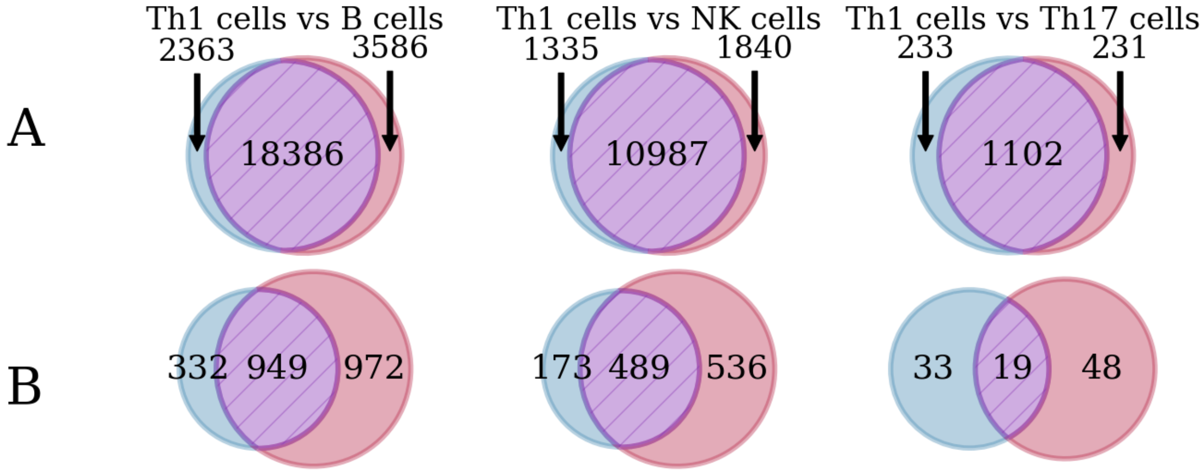

SFigure 3: Comparison of DEGs called on strand-aware and strand-unaware counts (RSEM+edgeR analysis pipeline). *Figure supplements Fig.3 of the main text but is implemented using alternative analysis tools. Three columns of Venn diagrams correspond to three comparisons of cell types indicated on top. Left circle (blue) corresponds to "true" DEGs identified with strand-aware counts. Row* ***A*** *shows overlap of DEGs obtained with strand-aware vs. strand-unaware counts. Row* ***B*** *is the same but only for strandedness-affected genes (those where log2(Nustno/Nstno) > 1).*

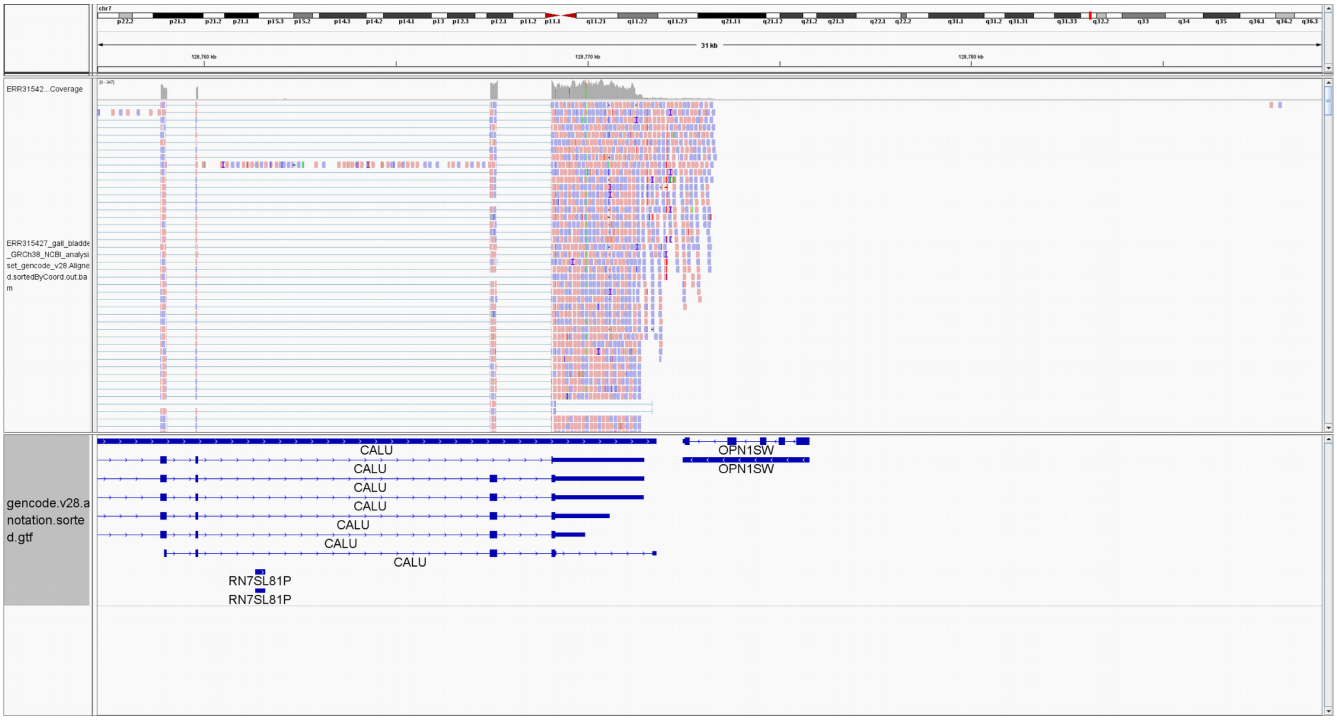

SFigure 4: IGV snapshot for read alignments in OPNSW1 gene locus. *Alignment track corresponds to gallbladder RNA-seq data from HPA. Note large number of reads which overlap leftmost exon of OPSIN gene but actually result from expression of CALU (calumenin) gene. Also note lack of reads spanning known splice junctions of the OPN1SW gene.*

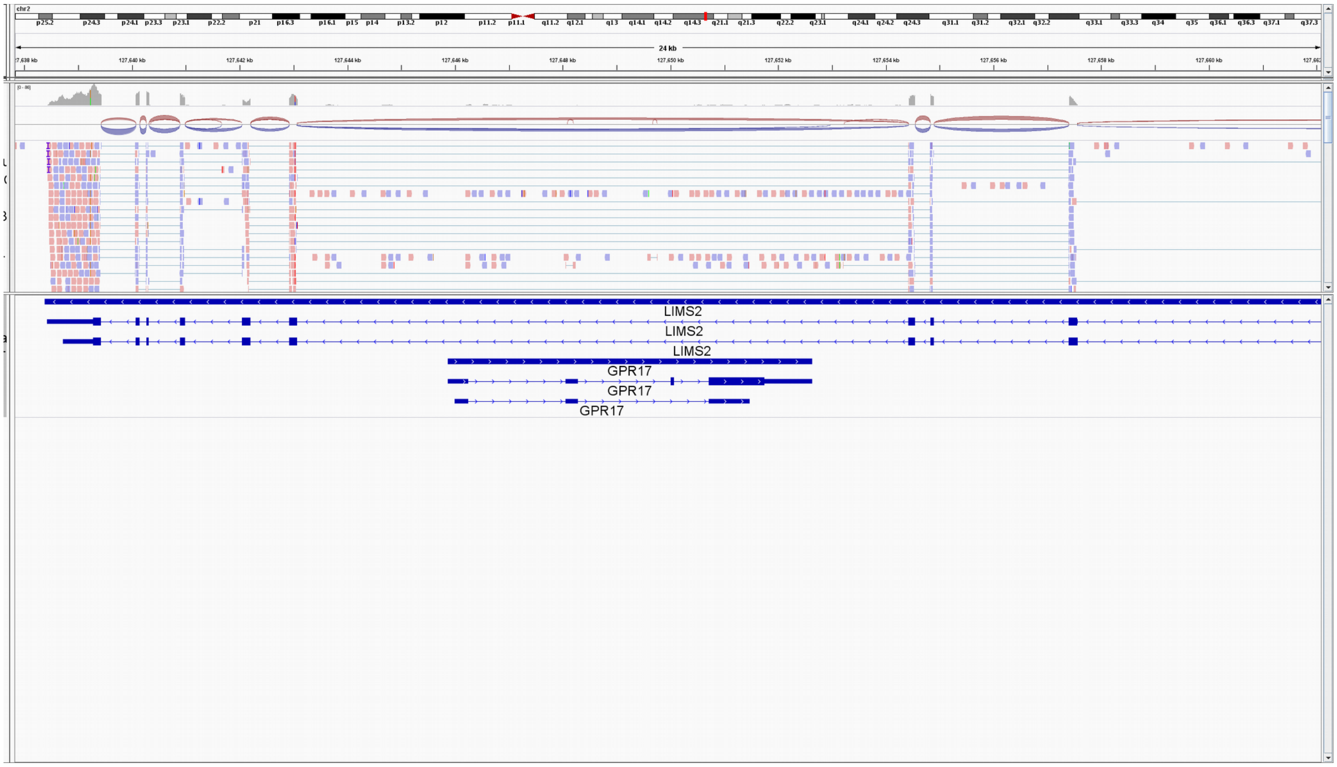

SFigure 5: IGV snapshot for read alignments in GPR17 gene locus. *Read alignment track corresponds to lung RNA-seq data from HPA. GPR17 gene is located on the opposite strand of LIMS2 gene within intronic region of the latter. Note that many reads overlapping GPR17 exons can as well emerge from nascent RNA of LIMS2 gene. Also note lack of reads spanning known splice junctions of the GPR17 gene.*

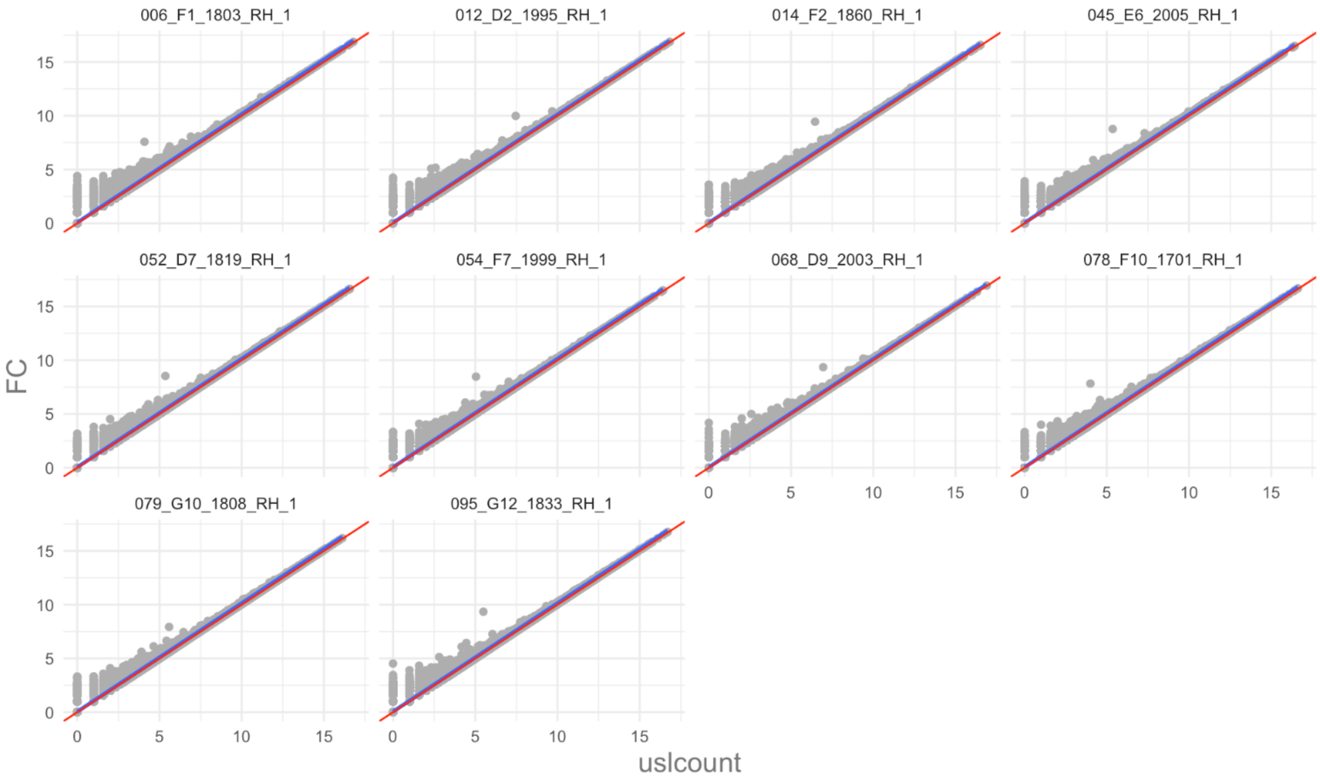

SFigure 6: featureCounts vs uslcount. *Comparison of counts obtained with featureCounts and uslcount. 10 Treg samples are used for generating the plot.*

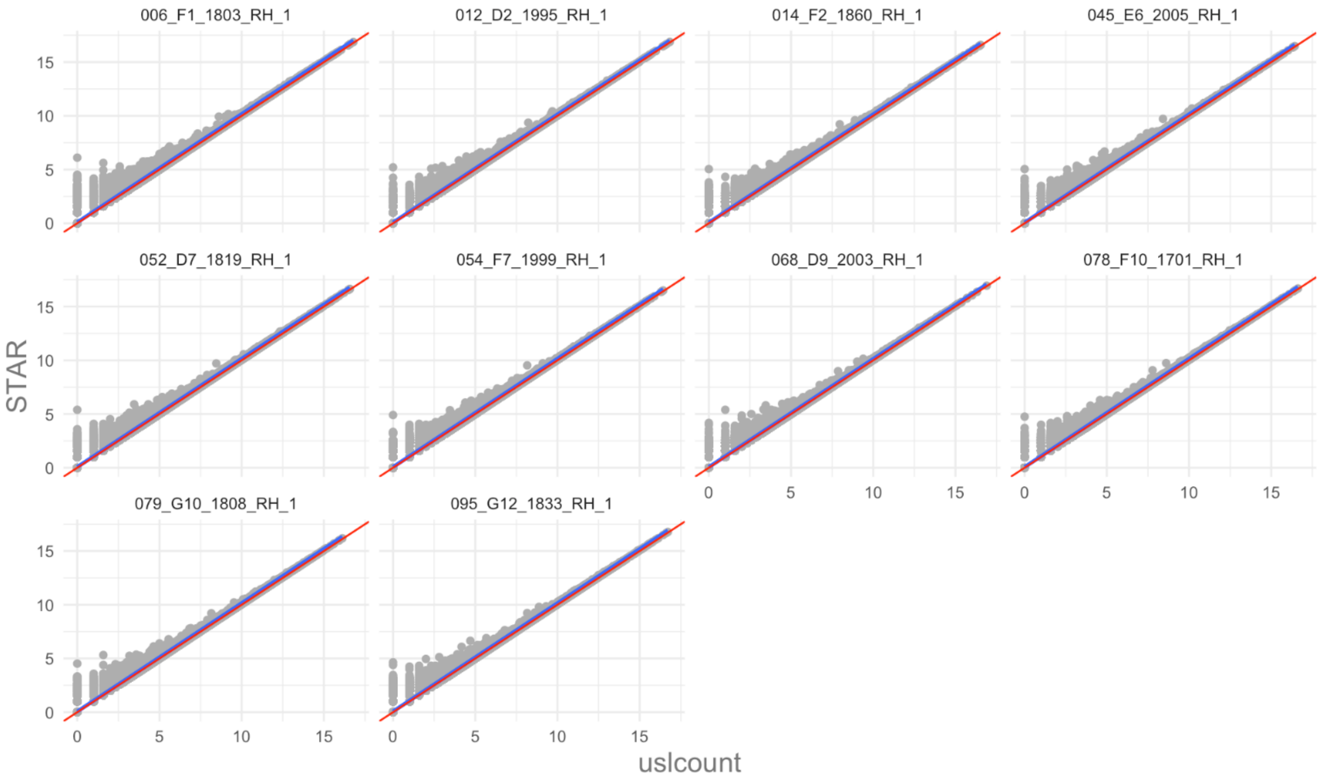

SFigure 7: STAR (counting function) vs uslcount. *Comparison of counts obtained with featureCounts and uslcount. 10 Treg samples are used for generating the plot.*

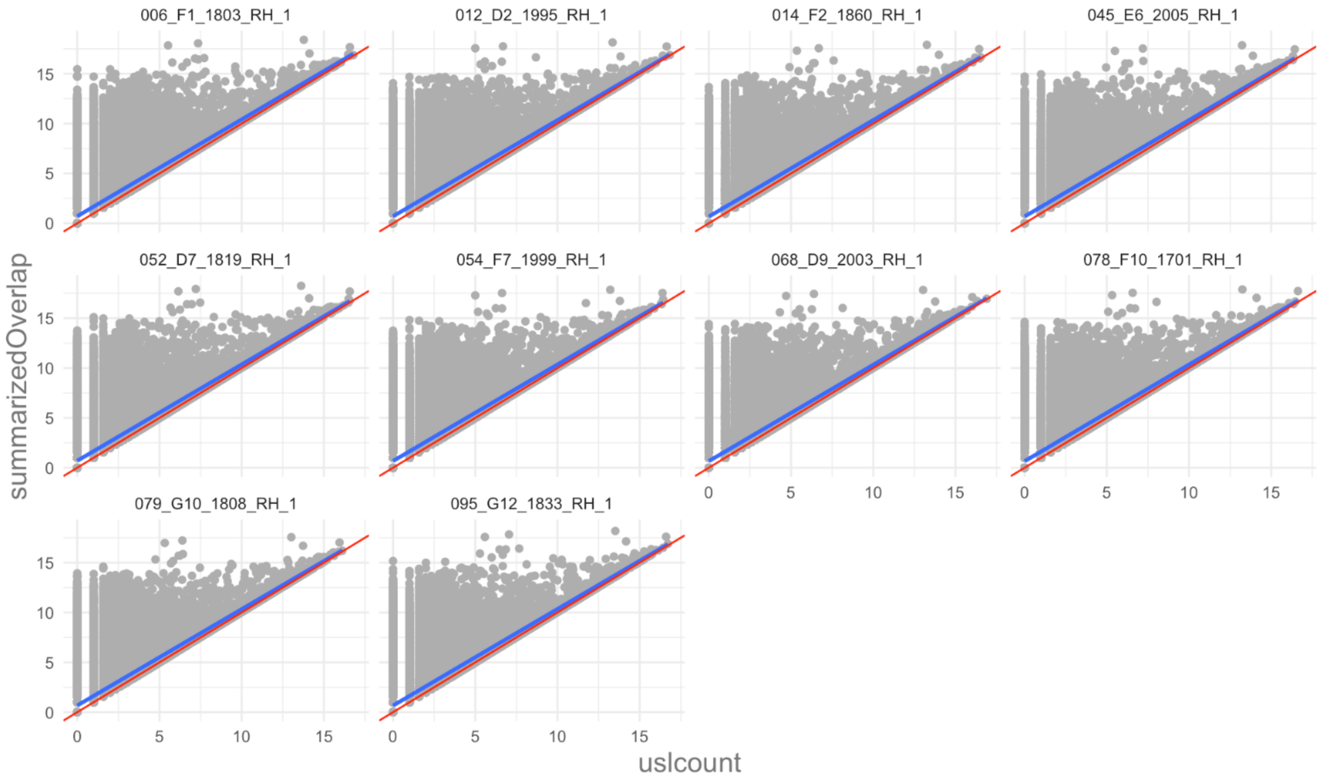

SFigure 8: summarizedOverlap (counting function) vs uslcount. *Comparison of counts obtained with summarizedOverlap and uslcount. 10 Treg samples are used for generating the plot.*

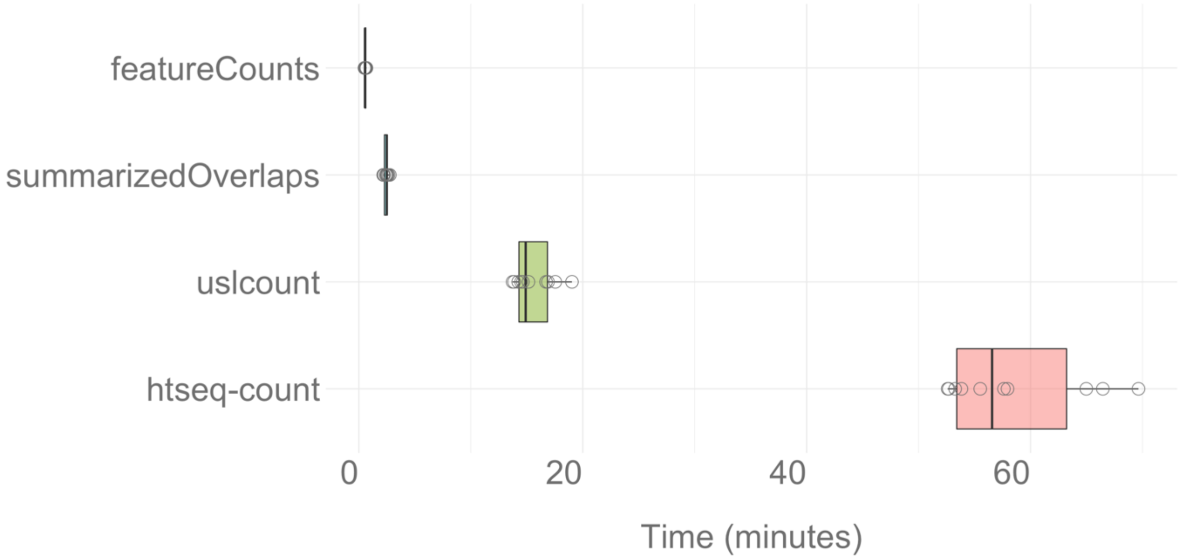

SFigure 9: Time charting for read counting softwares. *Comparison of time taken by indicated read counting softwares for 10 Treg samples.*

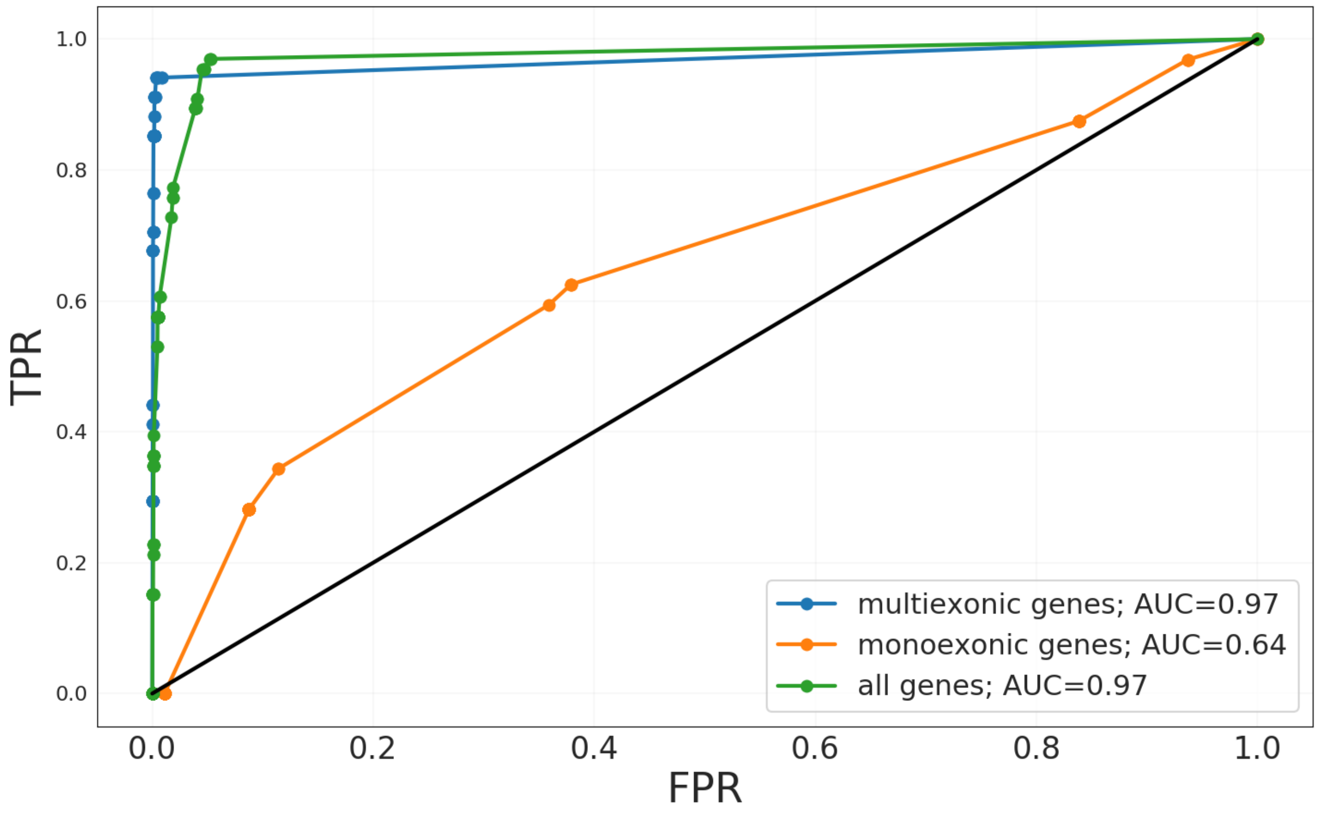

SFigure 10: ROC curves for paired-end data. *ROC curves were built for prediction of strandedness-affected genes in 75bp paired end dataset.*
